# Disseminated blastomycosis presenting as chronic verrucous lesions

**DOI:** 10.1016/j.idcr.2026.e02646

**Published:** 2026-06-19

**Authors:** Midila Bapineni, Naga Vamsi Krishna Machineni, Amna Khan, Rishika Kolluri, Maneesh Gaddam, Praveen Kumar Vikraman

**Affiliations:** a Appalachian Regional Healthcare, Department of Internal Medicine, Harlan, KY, USA; b University of Texas Southwestern Medical Center, Dallas, TX, USA; c Appalachian Regional Healthcare, Department of Pulmonary and Critical Care Medicine, Hazard, KY, USA; d Appalachian Regional Healthcare, Department of Infectious Diseases, Hazard, KY, USA

**Keywords:** Blastomycosis, Cutaneous blastomycosis, Disseminated blastomycosis, Blastomyces dermatitidis

## Introduction

Blastomycosis is a systemic fungal infection caused by the thermally dimorphic fungus *Blastomyces dermatitidis*, which is endemic to the Mississippi and Ohio River valleys and the Great Lakes region [1]. While pulmonary involvement is the primary clinical manifestation, hematogenous dissemination frequently occurs, most commonly targeting the skin. Because these cutaneous lesions often present as verrucous or ulcerative plaques, they can easily mimic malignancies, bacterial infections, or inflammatory dermatoses frequently leading to diagnostic delays. Activities involving exposure to disrupted soil and organic debris increase infection risk. We report a case of disseminated blastomycosis featuring extensive cutaneous involvement and asymptomatic pulmonary disease in a patient with poorly controlled diabetes.

## Case presentation

**Fig. 1:**
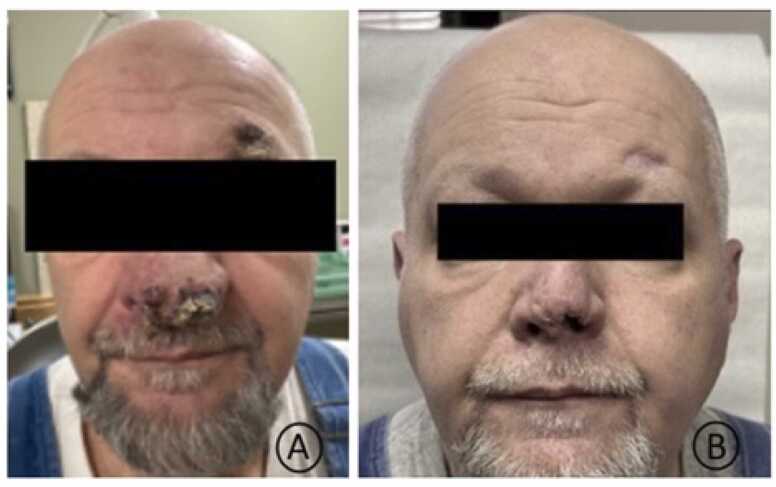
(A) Verrucous lesions involving the nasal tip and eyebrow at presentation. (B) Complete resolution of cutaneous lesions following six months of itraconazole therapy.

**Fig. 2:**
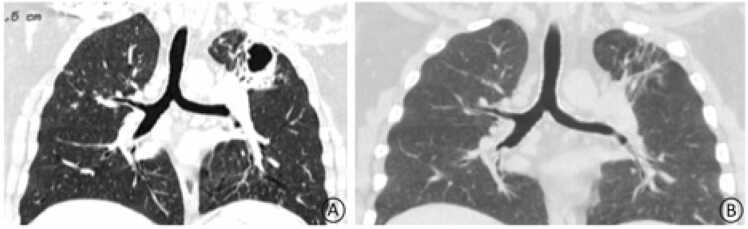
(A) CT chest demonstrating a 4.2 × 3.8 cm cavitary lesion in the left upper lobe. (B) Follow-up CT chest demonstrating marked interval improvement with only a small residual cavitary lesion remaining in the left upper lobe.

A 53-year-old Caucasian man with hypertension, hyperlipidemia, poorly controlled diabetes mellitus, coal worker's pneumoconiosis, and a history of logging and surface mining presented with multiple chronic, non-healing skin lesions involving the face, trunk, and extremities. The lesions had been present for approximately one year and were repeatedly treated as bacterial cellulitis or abscesses with several antibiotic regimens without improvement. Additional lesions subsequently developed on the left eyebrow and nasal tip, accompanied by intermittent epistaxis, generalized weakness, and a 15-pound unintentional weight loss. Physical examination revealed multiple verrucous and ulcerative plaques with raised violaceous borders and necrotic crusted centers (Fig. 1, Fig. 2 A). Laboratory testing showed mild anemia, elevated inflammatory markers, and poorly controlled hyperglycemia. HIV testing was negative. Urine *Blastomyces* antigen testing was positive, while *Histoplasma* antigen positivity was attributed to known cross-reactivity. Biopsies of facial lesions demonstrated granulomatous inflammation with broad-based budding yeast forms consistent with *Blastomyces dermatitidis* (Fig. 3A, Fig. 3B). Although the patient denied respiratory symptoms, computed tomography of the chest revealed multiple bilateral cavitary pulmonary lesions, confirming disseminated disease (Fig. 2 A). He was treated with oral itraconazole, resulting in complete resolution of cutaneous lesions (Fig. 1B) and marked radiographic improvement of pulmonary cavities after six months (Fig. 2B).

**Fig. 3A:**
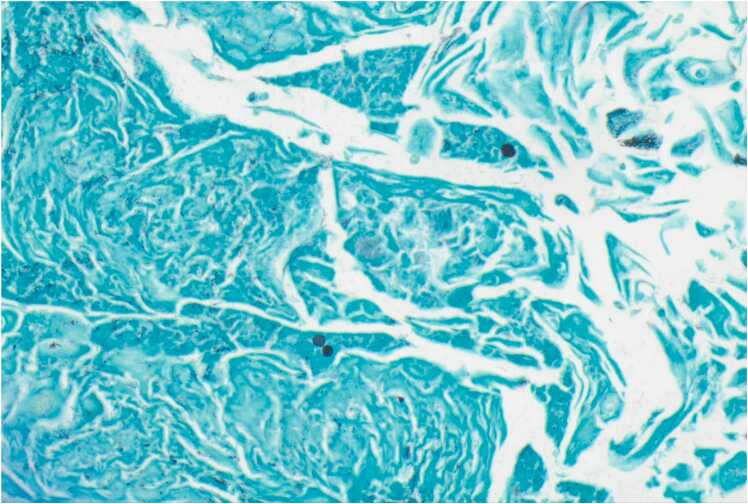
GMS stain demonstrating broad-based budding yeast.

**Fig. 3B:**
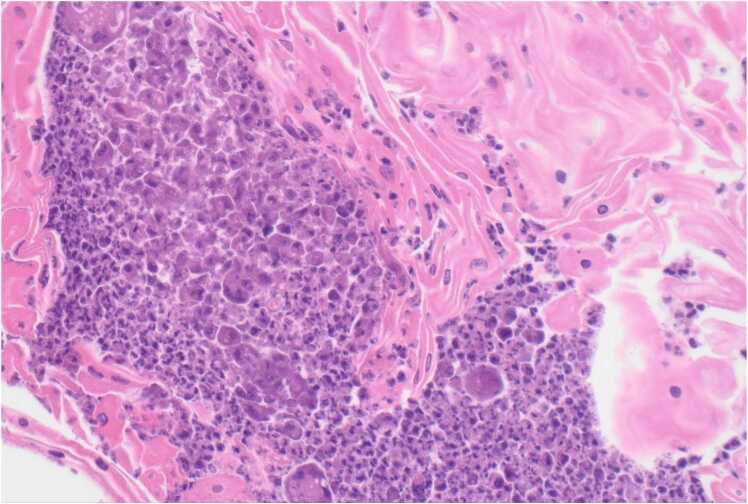
H&E stain demonstrating several multinucleated giant cells.

## Discussion

Blastomycosis remains a diagnostic challenge because of its ability to mimic infectious, inflammatory, and neoplastic conditions. This case emphasized the critical need for early clinical recognition of these cutaneous lesions and the need for thorough diagnostic evaluation.

## Author statement

The authors confirm that this manuscript is original, has not been published previously, and is not under consideration elsewhere. All authors have reviewed the manuscript, approved the final version, and agreed to its submission to IDCases.

## CRediT authorship contribution statement

**Naga Vamsi Krishna Machineni:** Writing – original draft, Investigation, Data curation, Conceptualization. **Amna Khan:** Writing – original draft, Investigation, Data curation, Conceptualization. **Rishika Kolluri:** Writing – review & editing, Conceptualization. **Maneesh Gaddam:** Writing – review & editing, Supervision, Data curation, Conceptualization. **Midila Bapineni:** Writing – original draft, Investigation, Data curation, Conceptualization. **Praveen Kumar Vikraman:** Writing – review & editing, Supervision, Investigation, Conceptualization.

## Ethical approval

Ethical approval was waived because this study is a case report, in accordance with local institutional policies.

## Consent

Written informed consent was obtained from the patient for publication of this case illustrated article and accompanying images. A copy of the written consent is available for review by the Editor-in-Chief of this journal on request.

## Funding

The authors received no specific funding for this work.

## Declaration of Competing Interest

The authors declare that they have no competing interests.
